# Who Should We Target? The Impact of Individual and Family Characteristics on the Expressed Need for Community-Based Treatment Support in HIV Patients in South Africa

**DOI:** 10.1371/journal.pone.0163963

**Published:** 2016-10-14

**Authors:** Edwin Wouters, Frederik le Roux Booysen, Caroline Masquillier

**Affiliations:** 1 Department of Sociology and Centre for Longitudinal and Life Course Studies, University of Antwerp, Antwerp, Belgium; 2 Centre for Health Systems Research and Development, University of the Free State, Bloemfontein, Republic of South Africa; 3 Department of Economics, University of the Free State, Bloemfontein, Republic of South Africa; University of New South Wales, AUSTRALIA

## Abstract

Reviews of impact evaluations of community-based health workers and peer support groups highlight the considerable variability in the effectiveness of such support in improving antiretroviral treatment (ART) outcomes. Evidence indicates that community-based support interventions targeting patients known to be at risk will probably display better results than generic interventions aimed at the entire population of people living with HIV. It is however difficult to identify these at-risk populations, rendering knowledge on the characteristics of patients groups who are in need of community-based support a clear research priority. The current study aims to address the knowledge gap by exploring the predictors of the willingness to (1) receive the support from a community-based health worker or (2) to participate in a support group in public sector ART programme of the Free State Province of South Africa. Based on the Individual-Family-Community framework for HIV research, the study employs a comprehensive approach by not only testing classical individual-level but also family-level predictors of the willingness to receive community-based support. In addition to individual-level predictors—such as age, health status and coping styles—our analysis demonstrated the importance of family characteristics. The results indicated that discrepancies in the family’s changeability level were an important predictor of the demand for community-based support services. Conversely, the findings indicated that patients living in a family more flexible than deemed ideal are more likely to require the support of a community health worker. The current study expands theory by indicating the need to acknowledge all social ecological levels in the study of chronic HIV care. The detection of both individual level and family level determinants of the expressed need for community-based support can inform health policy to devise strategies to target scarce resources to those vulnerable patients who report the greatest need for this support. In this way, the study results are a first step in an attempt to move away from generic, broad based community-based interventions towards community support that is tailored to the patient needs at both the individual and family level.

## Introduction

The introduction and subsequent widespread availability of public-sector antiretroviral treatment has transformed HIV/AIDS into a chronic illness. A study conducted by Bor and colleagues (2013) revealed a gain of 11.3 years in adult life expectancy—increasing from 49.2 in 2003 to 60.5 years by 2011 –since the implementation of the treatment [1]. A large-scale cohort study even demonstrated that South African HIV-positive adults can have a near-normal life expectancy, provided that they start ART early (at higher CD4 cell counts) [2, 3]–which is further supported by the country’s plans to shift to a Universal Test-and-Treat programme [4]. These favourable outcomes have led medical sociologists to conclude that—even in hard-hit countries like South Africa—HIV/AIDS has undergone a transition from a terminal illness to a controllable chronic condition, at least for those accessing treatment [5].

In order to replicate these positive findings in the long term, HIV care needs to be transformed into chronic disease care, i.e. with sufficient attention to the long-term psychosocial aspects of both the illness and its treatment. However, against the daunting challenge of providing additional psychosocial care to ever-growing patient groups, the question is increasingly raised “who will do the job?” [6]. The ART scale-up in sub-Saharan Africa, albeit successful, has clearly exposed the pre-existing weaknesses of the health systems in this region [7], in particular the inadequate supply and poor retention of skilled health professionals. As indicated by Hontelez and colleagues (2012), sustainable chronic disease treatment strategies thus require the mobilisation of additional human resources [8, 9].

Community mobilisation—community-based health workers and peer support groups in particular—has been increasingly cited as a feasible and durable response to chronic care needs associated with life-long ART in a context of severe shortages in the human resources for health [10–14]. Systematic review studies [13, 15] have assessed the impact of community-based interventions on a wide range of ART outcomes in resource-limited settings characterised by a high prevalence of HIV. According to the findings, support provided by *community health workers* could significantly improve ART outcomes in settings in which the tasks of healthcare professionals are usually limited to medical-technical services, in which the tasks of healthcare professionals are usually limited to medical-technical services—as demonstrated by recent studies in Malawi [16, 17], South Africa [18–22], Zambia [23] and Uganda [23–25]. With regards to *patient support groups* for people living with HIV, several studies have demonstrated a positive impact of this type of support on participants’ adherence levels [20, 26], physical health (CD4 cell count and viral load) [12, 27], and mental health (lower levels of perceived stigma and improved emotional well-being) [27–30].

Despite a clear general positive trend, review studies of the growing number of impact evaluations of community-based support programmes highlight the considerable variability in the effectiveness of such support in improving ART outcomes, ranging from non-significant effects to improvements of 20% and more [13, 15]. These discrepancies cannot only be attributed to measurement bias or the dissimilar characteristics of the various adherence intervention types under study, but are also related to the social contexts in which the community-based support initiatives are implemented. This suggests the need for research that incorporates these social, contextual reality in the assessment of community-based interventions [15].

Barninghausen et al. (2011) indicated that current scientific evidence is largely based on generic interventions aimed at the entire population of PLWHA while evidence from the developed world indicates that community-based support interventions targeting patients known to be at risk will probably display better results than untargeted interventions. However, the review also stipulates that it is difficult to identify these at-risk populations in sub-Saharan Africa, rendering knowledge on the characteristics of patients groups who are in need of and would thus like community-based treatment support a clear research priority.

Especially knowledge on the patient perspective is vital as undesired support will not produce the expected positive outcomes or might even produce negative results. In this regard, the recently developed Individual-Family-Community (IFC) framework stipulates that a crucial role could be played by the family context in which a community-based support initiative is introduced [31]. This was demonstrated in a recent study by Wouters et al. (2014) showing that a community-based peer adherence support intervention produced a positive impact on health in well-functioning families but a negative impact in ill-functioning families [32]. Special attention should thus be devoted to the question whether differing levels of family functioning could render family members with HIV more or less amenable to community-based adherence support.

The current study aims to address these research gaps by exploring the predictors of the willingness to (1) receive the support from a community-based health worker or (2) participate in a patient support group in patients enrolled in the public-sector ART programme of the Free State Province of South Africa. The study aims to employ a comprehensive approach by not only testing classical individual-level (age, gender, health, coping behaviour, stigma) but—in line with the IFC framework—also family-level predictors of the willingness to receive the above-mentioned two types of community-based support. In this way, the current study explicitly aims to apply the individual-family-community (IFC) framework for comprehensive HIV research which theoretically links the individual-, family- and community-level determinants of HIV outcomes [31]. Given the above-mentioned scientific evidence of the importance of the family level in explaining the potential impact of community-based interventions, we apply self-discrepancy theory to explicitly investigate whether discrepancies between the actual and ideal family situation increase the perceived need for community-based support.

## Methods

The current study aims explore the predictors of the willingness to receive community-based support by performing a secondary statistical analysis of post-trial data of the Effectiveness of Aids Treatment and Support in the Free State (FEATS) study conducted in the public-sector ART programme of the Free State Province of South Africa.

### FEATS study

The ‘Effective AIDS Treatment and Support in the Free State’ (FEATS) study, a three-year prospective cohort study conducted by the Centre for Health Systems Research and Development of the University of the Free State (UFS) was approved by the Ethics Committee of the UFS Faculty of Health Sciences [ETOVS 145/07 DOH-27-0907-2025] and is registered in the trial register of the National Institutes of Health [NCT00821366]. The study has two aims, namely, (a) to investigate the benefits of ART to patients, to the family members of patients on ART, and to communities at large and (b) to investigate the impact of a peer adherence support and a nutritional intervention on measures of treatment success—studied in a randomized controlled trial assigning patients across three study arms: those receiving standard care, those receiving additional peer adherence support; and those receiving both peer adherence and nutritional support. A Zelen-type double randomized consent design was adopted in the RCT component of the study [33]. Within such design, study participants are only offered the treatment to which they are randomized and can accept or reject treatment.

In order to yield statistically significant outcomes, 653 study participants were recruited into the study from 12 public ART clinics across five districts in the Free State Province of South Africa in 2007/08. Inclusion criteria included a minimum age of 18 years, having commenced ART within the past five weeks and residing in the town or village in which the particular health facility was located. Data collection at pre-trial baseline and at post-trial follow-up (two waves—on average 464 and 776 days after the baseline interview), comprised a patient interview and a household interview, inclusive of an adult questionnaire, conducted by trained enumerators using a structured questionnaire, in all cases only after written informed consent was obtained from study participants. The two follow-up waves yielded 498 (Wave 1) and 435 (Wave 2) completed interviews, respectively. Survey attrition was primarily due to mortality among study participants (42.4%) and unknown whereabouts (34.1%). A full description of the randomized controlled trial can be found in the CONSORT (CONsolidated Standards Of Reporting Trials) statement (including checklist and flowchart) of the overarching FEATS study added to the article as a supplementary file. The current study employs data for the 435 patients interviewed at both follow-up waves as data on family functioning was not collected at baseline.

### Study variables

As this is the first study to explore the determinants of expressed need of ART patients for different types of community-based support using the IFC framework [31], we selected a wide range of individual-level (demographic, health-related, and psychosocial) and family-level variables that have been significantly associated with the well-being in HIV patients on ART, and thus consequently with patients’ willingness and need for the support of community-based lay health workers and support groups [34–39]. Data on all potential predictors (except for the relatively stable demographic data) was gathered at two time points (Wave 1 & 2).

First of all, we introduced a number of demographic variables in the analysis. Data on *age*, *sex* and *educational level* were collected [34–37]. Educational level was measured according to five categories: no education, primary education, some secondary education, matric education (grade 12) or tertiary/post-matric education.

In addition and in accordance with the literature, we included health-related variables, namely patients’ self-reported health status and self-reported adherence. The health related variables were introduced as we hypothesize that patients who struggle with their treatment and/or have poorer health require the treatment-related support of lay health workers or support groups more than adherent and stable patients. Patients’ self-reported health status was measured by the *EUROQoL Visual Analogue Scale*. Self-reported adherence was measured using the *Center for Adherence Support Evaluation (CASE) Adherence Index*, composed of three simple questions addressing three different aspects of ART adherence: difficulty taking ART medication on time, frequency of missed ART doses and time since most recent missed ART dose. The index has been shown to correlate strongly with the three-day recall method, and to predict virologic and immunologic response [40].

As community-based support services not only offer treatment support but also focus on the mental health of patients, we have included two psychosocial variables—HIV-related stigma and different coping styles—in our analyses. The stigma scale was generated from eight items in the questionnaire, asking respondents about the extent to which they agreed or disagreed with perceptions that they or others had regarding HIV and AIDS. Two dimensions of stigma were measured: *external stigma* (5 items), reflecting the negative social identity ascribed to PLWHA by other people, and *internalized stigma* (3 items), which occurs when external stigma is internalized by the patient resulting in negative self-image, feelings of shame or guilt, and other manifestations of felt stigma [41]. Both types of stigma were operationalized using an adaptation of Berger’s HIV stigma scale presented by Wright et al. [42]. Two coping behaviors were measured: *positive* and *social support seeking coping styles*. Respondents were asked to describe how they were currently dealing with living with HIV and AIDS by answering ‘yes’ or ‘no’ to a series of statements taken from a study conducted in the United States. In this study, we included two sub-scales identifying ‘positive’ and ‘seeking social support’ as the two most relevant coping mechanisms of people with AIDS [43]. Both the stigma and coping scales were previously successfully applied in studies on the mental health of HIV patients on ART in South Africa [38].

Given the fact that the current study employs trial data, we included a variable representing the peer adherence support intervention of the trial. Peer adherence support comprised bi-weekly visits by a trained *community-based peer adherence supporter* who at recruitment had been on ART for at least 12 months. Recruited peer adherence supporters were provided with five days of theoretical and practical training on antiretroviral treatment and adherence support. Peer adherence supporters were paid a monthly stipend of ZAR 800 and were required to pay two visits each week to eight ART patients over a period of 18 months. The peer adherence supporters performed a wide range of adherence counselling tasks.

As previous studies have indicated the importance of the family level in explaining the differential impact of community-based support in different contexts [32], the current study explicitly incorporates a number of family-related variables. In accordance with standard practice in national household surveys, a family is defined as the patient and those individuals who (a) lived under the same ‘roof’ or within the same structure at least four nights per week out of the past month, (b) share food from a common source when they are together, i.e. eat together, and (c) contribute to or share in the common resource pool.

Based on a combination of the IFC framework and discrepancy theory, the current study hypothesizes that the perceived inability of the family to provide the psychosocial support required to durably adhere to treatment predicts the reported willingness to receive support from outside the family—i.e. from a community-based lay health worker and/or a patient support group. Family resiliency theory states that levels of intra-family functioning play a crucial role in fostering individual well-being and health, especially in a context of disruptive life challenges (in this case, life with HIV as a chronic illness) [44–46]. Within this framework, discrepancy theory predicts that discrepancies between the actual level of family functioning and the ideal level of family functioning (i.e. representations of an individual’s beliefs about his or her wishes and aspirations for the family) are associated with a greater perceived need for community-based support [47, 48]. In other words, the discrepancy between the actual and ideal family situation determines whether support from outside the family (i.e. community-based initiatives such as lay health workers and support groups) is desired.

The Family Attachment and Changeability Index 8 (FACI-8), developed by McCubbin, Thompson and Elver (1996), was used to measure (discrepancies in) family functioning [49]. According to the above-mentioned family resiliency model, the outcomes of all family dynamics result in a certain degree of adaptation and functioning in the family. The culturally and ethnically sensitive FACI-8 is a 16-item scale designed especially to measure levels of family functioning, using two subscales to assess Attachment and Changeability [49]. The Attachment subscale consists of eight items (e.g. “In our family, everyone goes his/her own way” and “We have difficulty thinking of things to do as a family”). The subscale was designed to ascertain the strength of the family members’ attachment to each other. The Changeability subscale consists of eight items that determine the relative flexibility of family members in their relationships with each other (e.g. “Our family tries new ways of dealing with problems” and “Each family member has input in major family decisions”).

At each time point, the 16 FACI-8 items were asked twice to assess (1) the *actual* family situation (i.e. the actual attachment and changeability scores) as well as (2) the *ideal* family situation (i.e. the attachment and changeability scores considered to be ideal according to the respondent). In accordance with the family resilience model [50], the ideal attachment and changeability scores are subtracted from the ideal attachment and changeability scores in order to compute categorical variables at each time point describing whether the perceived level of attachment or changeability in the family (1) surpasses, (2) equals or (3) is lower than the ideal level of attachment or changeability. In order to incorporate these categorical variables with three categories, we computed—for each discrepancy variable—two (number of categories minus one) dummy variables, (1) one distinguishing the respondents who reported a lower actual attachment or changeability level than their ideal level (value 1) from those respondents who reported the actual and ideal to be identical and those who reported higher attachment/changeability levels than the ideal level (value 0); (2) the other distinguishing the respondents who reported a higher actual attachment or changeability level than their ideal level (value 1) from those respondents who reported the actual and ideal to be identical and those who reported lower attachment/changeability levels than the ideal level (value 0). The category that is not coded (ideal and actual are equal) thus becomes the category to which the other two categories will be compared. Consequently, we end up with eight dummy variables: (1) higher actual changeability score than ideal at wave 1; (2) higher actual attachment score than ideal at wave 1; (3) lower actual changeability score than ideal at wave 1; (4) lower actual attachment score than ideal at wave 1; (5) higher actual changeability score than ideal at wave 2; (6) higher actual attachment score than ideal at wave 2; (7) lower actual changeability score than ideal at wave 2; and (8) lower actual attachment score than ideal at wave 2.

In order to assess the true effect of discrepancies in family functioning the need for community-based support, two additional family-related confounders were included in the regression analysis: (1) *household size*, a continuous variable measuring the total number of persons living in the household and (2) a measure of the family’s socio-economic position, namely the *real per capita monthly household income* (standardized) as increasing evidence demonstrates that there is a strong link between household level income and individual health and wellbeing, especially in vulnerable patient groups [51, 52].

Finally, the willingness to be visited by a community-based lay health worker was measured by the question “Would you like to have a community or lay health worker visit you at home?”. The willingness to participate in a peer support group was measured with the question “Would you like to participate in a support group for people living with HIV/AIDS and/or people on ARV treatment?” Naturally, the small proportion of patients receiving the support of a CHW (14.5%) or participating in a support group (7.1%) were considered to display a willingness to receive this support as they did not only express their willingness but also acted on it. The limited sample size (and wide range of predictors) meant that we could only include the data on these two dependent variables gathered at wave 2.

### Data analysis

Cross-lagged regression analyses were used to assess the relationship between a range of individual-level (demographic, health-related, psychosocial) and family-level predictors—measured at two time points—and the willingness to (1) be visited by a community-based lay health worker and (2) participate in a peer support group. Modelling was carried out using robust weighted least squares (WLSMV) in the Mplus program version 5 (www.statmodel.com). The Mplus estimates for paths from predictors to an observed categorical dependent variable (such as (a) the willingness to be visited by a CHW or (b) participate in a support group)) are probit regression coefficients (Bs). A positive sign means that the probability of the categorical dependent variable (e.g. the category 1 for a 0/1 variable) is increased when the predictor value increases. A larger magnitude means that this probability is higher. The cross-lagged model contains three types of regression paths. A first type connects like variables over time and thus represents within-variable regression paths (stability paths). By including regression paths between the same variable measured in different survey waves, we can estimate its relative stability across time. Secondly, the model contains the standard regression paths from individual and family-level variables on the willingness to accept support. Finally, we included cross-lagged effects (between Wave 1 and 2) of all time-variant predictors on the willingness to accept support of a CHW or support group to test the impact over time. The WRMR (Weighted Root Mean Square Residual) and Root Mean Square Error of Approximation (RMSEA) are used as a model fit index.

## Results

### Sample description

The characteristics of our sample are depicted in Table 1.

**Table 1:** Sample characteristics (n = 435).

	Wave 1	Wave 2
Age (mean ± SD)	39.1 ± 9.1	
Sex		
Male	22.7	
Female	77.3	
Education (%)		
No formal education	3.4	
Primary education	26.0	
Some secondary education	47.6	
Grade 12 / matriculation	20.0	
Tertiary education	3.1	
Peer adherence intervention (%)	85.1	
CASE ^1^	15.1 ± 2.3	15.5 ± 1.8
Health status (EQ-VAS) ^2^	81.8 ± 17.3	82.1 ± 16.5
Positive coping (mean ± SD) ^3^	4.9 ± 0.4	4.9 ± 0.4
Avoidant coping (mean ± SD) ^4^	4.1 ± 1.7	3.8 ± 1.5
Social support seeking coping (mean ± SD) ^5^	1.5 ± 0.7	1.7 ± 0.6
External stigma (mean ± SD) ^6^	11.3 ± 2.9	11.5 ± 2.8
Internal stigma (mean ± SD) ^7^	4.5 ± 1.9	4.4 ± 1.8
Per capita household income (ZAR, mean ± SD)	588.6 ± 609.5	830.5 ± 3000.2
Household size (mean ± SD)	3.2 ± 1.9	3.1 ± 1.9
Family functioning (FACI-8)		
Attachment		
Higher than ideal at Wave (%)	34.0	21.3
Lower than ideal at Wave (%)	25.5	34.0
Changeability		
Higher than ideal at Wave (%)	6.9	8.3
Lower than ideal at Wave (%)	50.7	48.1

^1^ The CASE adherence index ranges between 3 and 16 (higher values denoting better adherence)

^2^ The EQ-VAS asks patients to indicate their overall health on a vertical visual analogue scale (a 20 com vertical line), ranging from “worst possible” (0) to “best possible” health (100).

^3^ The Positive coping scale ranges from 0 to 5 (higher values denoting more positive coping)

^4^ The Avoidant coping scale ranges from 0 to 9 (higher values denoting more avoidant coping)

^5^ The social support seeking coping scale ranges from 0 to 2 (higher values denoting more social support seeking coping)

^6^ The external stigma scale ranges from 5 to 20 (higher values denoting more stigma)

^7^ The internal stigma scale ranges from 3 to 12 (higher values denoting more stigma)

The overall model testing the relationship between a wide range of individual-, family- and community-level variables and the expressed need to (a) receive the support of a community health worker and (b) participate in a support group displayed an acceptable fit with an RMSEA of 0.037 and a WRMR of 1.083.

### Community health worker

Table 2 shows the results of the probit regression explaining the willingness to receive the support of a CHW. None of the demographic variables was significantly associated with the demand for the support of a community health worker. Two psychosocial variables were significantly associated with the perceived need for the support of a community health worker. Patients displaying positive coping strategies at Wave 2 were significantly less likely to express the need for CHW support (β = -0.237, P = 0.002). Similarly, patients displaying the seeking-social-support-coping style were significantly more likely to report a willingness to receive the support of a CHW (β = 0.220, P = 0.001). With regards to the family-level variables, high levels of changeability—exceeding the ideal level—seem to be positively associated with the expressed need for CHW support. Patients who reported that their family was more flexible or democratic than their ideal family at both Wave 1 (β = 0.253, P = 0.024) and 2 (β = 0.248, P = 0.047) were significantly more likely to report a willingness to receive the support of a CHW: living in such a family context increases the predicted probability of desiring the support of a community health worker. Finally, the bi-weekly visits by a trained community-based peer adherence supporter as part of the trial’s intervention significantly increased predicted probability of wanting the support of a CHW at Wave 2 (β = 0.190, P = 0.012).

**Table 2:** The relationship between individual-level, family-level (including attachment), and community-level variables and the willingness to receive support from a community health worker or participate in a support group: standardized probit regression coefficients (minus relative stability paths) (n = 435).

	Community health worker			Support group		
	Estimate (B)	Standardized estimate (β)	P-value	Estimate (B)	Standardized estimate (β)	P-value
**Individual**						
Age	0.015	0.106	0.245	**-0.036**	**-0.273**	0.003
Sex	0.333	0.104	0.169	-0.018	-0.006	0.936
Education	0.116	0.074	0.402	-0.229	-0.158	0.074
Health status Wave 1	-0.008	-0.104	0.173	**-0.011**	**-0.146**	0.015
Health Status Wave 2	0.012	0.144	0.071	0.005	0.066	0.392
Adherence (CASE) Wave 1	0.042	0.075	0.400	-0.024	-0.047	0.621
Adherence (CASE) Wave 2	0.002	0.002	0.970	-0.044	-0.049	0.332
Positive coping Wave 1	-0.071	-0.019	0.809	0.368	0.106	0.164
Positive coping Wave 2	**-0.997**	**-0.237**	0.002	**-0.544**	**-0.140**	0.004
Social support seeking coping Wave 1	0.070	0.038	0.651	0.021	0.012	0.890
Social support seeking coping Wave 2	**0.461**	**0.220**	0.001	0.055	0.028	0.702
External stigma Wave 1	0.006	0.012	0.212	0.063	0.145	0.062
External stigma Wave 2	-0.050	-0.102	0.141	**-0.098**	**-0.200**	0.014
Internal stigma Wave 1	0.087	0.120	0.109	0.007	0.010	0.898
Internal stigma Wave 2	-0.012	-0.016	0.847	-0.006	-0.009	0.905
**Family**						
Per capita household income Wave 1	0.104	0.074	0.461	**0.268**	**0.207**	0.036
Per capita household income Wave 2	-0.252	-0.253	0.179	-0.239	-0.258	0.090
Household size Wave 1	-0.061	-0.091	0.280	0.068	0.111	0.150
Lower attachment Wave 1	-0.002	-0.001	0.996	-0.030	-0.011	0.920
Lower attachment Wave 2	0.176	0.138	0.201	0.150	0.127	0.239
Lower changeability Wave 1	0.259	0.097	0.375	**0.509**	**0.207**	0.040
Lower changeability Wave 2	0.015	0.011	0.916	0.113	0.095	0.366
Higher attachment Wave 1	-0.036	-0.013	0.907	-0.003	-0.001	0.991
Higher attachment Wave 2	0.010	0.008	0.943	0.008	0.006	0.957
Higher changeability Wave 1	**1.131**	**0.253**	0.024	0.409	0.099	0.300
Higher changeability Wave 2	**0.329**	**0.248**	0.047	0.037	0.031	0.826
**Community**						
Peer adherence support intervention	**0.685**	**0.190**	0.012	0.021	0.006	0.940
**RMSEA** ^1^		**0.037**			**0.037**	

^1^ Root Mean Square Error of Approximation

### Peer support group

Age was the only demographic variable to be significantly but negatively associated with the willingness to participate in a peer support group (see Table 2). Older age decreased the predicted probability of admission. For a one unit increase in age, the z-score decreased by 0.036 (β = -0.273, P = 0.003). A patient’s health status was also significantly associated with the perceived need for the participation in a peer support group: healthier patients were less likely to require the support of peers (β = -0.146, P = 0.015). Two psychosocial variables were significantly associated with the demand for a peer adherence support group. Patients displaying positive coping strategies at Wave 2 were significantly less likely to want to participate in a support group at Wave 2 (β = -0.140, P = 0.004). Similarly, the experience of external stigmatizing attitudes at Wave 2 decreased the likelihood to want to participate in a support group at the same point in time (β = -0.200, P = 0.014). With the regards to the family-level variables, we observe two significant associations. Patients who reported that their family was less flexible than their ideal family were significantly more likely to report the willingness to participate in a patient support group: living in such a family context increases the Z-score of the support group variable with 0.509 (β = 0.207, P = 0.040). Family’s economic status, measured by the standardized real per capita monthly household income at Wave 1 was positively associated with the willingness to participate in a peer support group at Wave 2 (β = 0.207, P = 0.037).

## Discussion

The aim of the current study was to explore the predictors of the willingness to (1) receive support from a community health worker or (2) to participate in a patient support group in a sample of HIV patients in the public-sector ART programme of the Free State Province of South Africa. Based on the IFC framework for HIV research [31], the study employed a comprehensive approach by not only testing classical individual-level (age, gender, health, coping behaviour, stigma) but also family-level correlates of the willingness to receive the above-mentioned two types of community-based support.

The study results did not reveal strong links between the demographic characteristics of the patients interviewed and their willingness to receive the support of either CHWs or a peer support group. Age was the only variable to be significantly associated with the demand for community-based support, with older people being less likely to express the wish to participate in a peer support group. Similarly, patients’ health status was also not a strong correlate of the demand for extra support: patients self-reported health status was only significantly (and negatively) associated with the need to participate in a peer support group. The lack of a significant association between patients’ health status and the demand for the support of a CHW as well as the lack of an association between the adherence index and the demand for either of the two support services is worrying. Several studies have demonstrated the positive relationship between CHW as well as peer support on the one hand and ART adherence and resulting health outcomes on the other hand [11, 12, 14, 20, 24, 26, 31, 53, 54]. The fact that the most vulnerable patient groups—those with low levels of adherence and/or low levels of self-perceived health—do not express a greater need for additional support—especially from a CHW, stresses the need for more targeted interventions which are adapted to the special needs of these vulnerable patient groups.

Several psychosocial variables appeared to be solid correlates of the demand for community-based support initiatives. A positive coping strategy was associated with a lower expressed need for the support of a CHW or a patient support group at the same point in time. This is in line with previous studies demonstrating that positive coping strategies were related to higher levels of adherence [55] and better mental health [56], signifying the fact that these patients are adapting to their situation well and do not necessarily require additional support measures. In addition, the seeking social support coping style was associated with a higher probability of wanting the support of a CHW. This logical is in line with the views expressed by the developers of the seeking social support scale, Fleishman and Fogel (1994), who stated that seeking social support may result when distress reaches unacceptable levels, creating the need for additional support of a CHW [43]. Our finding that patients’ external stigma was negatively associated with the expressed willingness to participate in a peer support group suggests that expected negative reactions act as a barrier to potential participation in a peer support group. This is particularly worrying as research has assigned peer support groups where patients can share their experiences in the relatively safe company of peers as a potential answer to stigmatization [29]. These findings urge policy makers and support programs (1) to actively search for the most vulnerable patient groups, those in psychosocial distress and in fear of stigmatization who need community-based support most but are afraid of accessing it and (2) to move away from generic, broad-based interventions as these do not reach the most vulnerable patient groups nor do they necessarily facilitate treatment adherence within these patient groups—a recent study by Wouters et al. (2014), for example, demonstrated a positive impact of a generic support initiative in well-functioning families and a negative impact in more vulnerable, ill-functioning families [32]. Community support programs should thus provide support that is sensitive to the suboptimal contexts in which they should often be implemented.

Previous studies have indicated the importance of the family level in explaining the differential impact of community-based support in different contexts [28, 32]. The current study applied self-discrepancy theory to investigate whether discrepancies between the actual family situation and ideal family situation increase the perceived need for community-based support. The results indicate that discrepancies in the family’s changeability level are an important correlate of the demand for community-based support services. Patients reporting at Wave 1 that their family is not flexible enough in their relations with each other have a higher probability of wanting the support of a peer support group at Wave 2 than patients living in a family of which the actual changeability level equalled the ideal level. These results indicate that living in a rather rigid family where the members do not have the sense that they can actively participate in the decision process when reacting to a crisis, are more likely to seek help outside the family unit. Conversely, the findings indicated—cross-sectionally as well as cross-lagged—that patients living in a family which is actually more flexible than deemed ideal are more likely to require the support of a community health worker. It could be argued that these patients would welcome a stabilizing factor in their unsteady family situation. These patients feel that they bear a (too) large responsibility in making (health-related) family decisions and would like the support of a CHW. In other words, patients living in rigid families seek help outside in the more like-minded context of a peer support group while patients living in an over-flexible or over-democratic environment would welcome the home visits of a CHW as a steady supportive mechanism. These results are in line with earlier findings that the receptivity for and resulting efficacy of a peer adherence intervention is highly dependent upon the family context in which it is implemented [28, 32]. As this is—to the best of our knowledge—the first study to actively research the association between family dynamics and the demand for community-based support, more research is needed to further ascertain the intricate links between family dynamics and the need for additional outside support.

Finally, we found a positive association between the peer adherence intervention of the trial and the welcoming of CHW visits in the future. This result indicates that the patients valued the support of the peer adherence support intervention provided by the experienced peer adherence supporters and maybe saw the availability of a CHW as a valuable substitution for the support that ended with the end of the trial. Previous studies have reported the fact that patients and their families value the emotional, instrumental or informational support provided by community-based supporters which could explain the higher demand by community-support-experienced patients compared to patients who did not enjoy the benefits of the trial’s peer adherence support [13, 15].

It should be noted that our analyses discovered both cross-sectional and cross-lagged associations. The results showed that the different types of coping behaviours as well as the external stigma experienced were significantly correlated at Wave 2, displaying an association of a cross-sectional nature. The majority of the cross-lagged paths (health status, household income, lower changeability) emerged in the analysis explaining the willingness to participate in a patient support group. Only one variable (higher changeability) was significantly associated with the willingness to receive CHW support at both time points (both cross-sectionally and cross-lagged), all other significant relationships were cross-sectional. It is, however, difficult to explain these discrepancies. One potential explanation is that introduction into a peer support group—and thus the opening up to a group of strangers—seems not feasible or desirable in the midst of a crisis but needs to be initiated first in times of relative stability in order to gradually build trust and gain confidence in the group. This support could then be useful in subsequent crises. However, further in-depth, qualitative research is needed to disentangle these interrelationships and provide more insight into the mechanisms underlying the willingness to receive CHW support and participate in a peer support group.

The strengths of this study include its theoretical foundation in self-discrepancy theory and family sociology as well as the availability of longitudinal information on a range of individual level and family level determinants of the demand for support in a sample of ART clients in South Africa. To the best of our knowledge, ours is the first study to apply the IFC framework to comprehensively study the demand for community-based support initiatives (patient support groups and CHWs) in a resource-limited setting characterised by a high prevalence of HIV [31]. Our study is nevertheless subject to several limitations. First of all, respondents were drawn only from those HIV infected individuals who had gained access to the public sector antiretroviral treatment program and had successfully completed drug-readiness training. Patients in need for community-based support—be it from a support group or from a CHW—may be less likely to seek care, complete drug readiness, and initiate treatment. They are thus under-represented in this study and one can thus only compare our findings with other patient groups who have accessed ART. Secondly, our study employed a large dataset containing information on a wide range of relevant individual-level and family-level aspects of life with HIV in South Africa. However, this strategy did not allow us an in-depth investigation of all these aspects. In order to truly disentangle the complex interrelationships between (1) determinants at the individual level, (2) all aspects of family life in a challenging context, and (3) the demand for community-based support, one would need to add a layer of in-depth qualitative investigation to the current more superficial quantitative study. More research is thus needed to study how and why psychosocial coping mechanisms and family dynamics impact the demand for additional community-based support. Thirdly, the authors want to stress that there is considerable variability in the community support initiatives active in the HIV programs in sub-Saharan Africa. In order to produce generalizable outcomes, researchers need to clearly define their roles and responsibilities. In the current study, for example, CHWs were trained, multipurpose HIV/tuberculosis workers, involved in counselling, adherence support and home-based care. A different CHW system—for example with CHWs receiving incentives for tracking defaulting patients—might result in different outcomes.

Several important insights have emerged from this study, with implications for both theory and practice. From a theoretical point of view, the combination of self-discrepancy theory and family sociology within the wider IFC framework draws attention to the role of the different layers of the IFC framework [31]. In addition to individual-level factors, future research should thus also incorporate family-level variables, as the demand for community-based support is interconnected with family dynamics. The current study thus expands the theoretical and conceptual scope of studies of this nature by indicating the need to acknowledge all social ecological levels in the study of chronic HIV care. The study findings can also have important implications for public health policy and practice. The detection of both individual-level and family-level correlates of the expressed need for community-based support can inform health policy to devise strategies to target the scarce resources to those vulnerable patients who report the greatest need for this support—i.e. patients displaying low levels of positive coping, high levels of seeking social support coping and discrepancies between the actual and ideal family situation. In this way, the study results are a first step in an attempt to move away from generic, broad based community-based interventions towards community support that is tailored to the patient needs at both the individual and family level. Additional longitudinal studies and qualitative research is however needed in order to provide full clarification of the mechanisms by which these individual and family determinants impact the need for community-based support initiatives in chronic HIV care.

## Supporting Information

S1 Dataset This is the FEATS dataset used to assess the impact of individual and family characteristics on the expressed need for community-based treatment support in HIV patients in South Africa. (SAV)
